# Representational Content of Oscillatory Brain Activity during Object Recognition: Contrasting Cortical and Deep Neural Network Hierarchies

**DOI:** 10.1523/ENEURO.0362-20.2021

**Published:** 2021-05-24

**Authors:** Leila Reddy, Radoslaw Martin Cichy, Rufin VanRullen

**Affiliations:** 1 Artificial and Natural Intelligence Toulouse Institute, Université de Toulouse 3, Toulouse 31052, France; 2 Centre National de la Recherche Scientifique, Centre de Recherche Cerveau et Cognition (CerCo), Toulouse 31052, France; 3 Department of Education and Psychology, Freie Universität Berlin, Berlin 14195, Germany

**Keywords:** brain oscillations, deep neural networks, fMRI, MEG, object recognition, representational similarity analysis

## Abstract

Numerous theories propose a key role for brain oscillations in visual perception. Most of these theories postulate that sensory information is encoded in specific oscillatory components (e.g., power or phase) of specific frequency bands. These theories are often tested with whole-brain recording methods of low spatial resolution (EEG or MEG), or depth recordings that provide a local, incomplete view of the brain. Opportunities to bridge the gap between local neural populations and whole-brain signals are rare. Here, using representational similarity analysis (RSA) in human participants we explore which MEG oscillatory components (power and phase, across various frequency bands) correspond to low or high-level visual object representations, using brain representations from fMRI, or layer-wise representations in seven recent deep neural networks (DNNs), as a template for low/high-level object representations. The results showed that around stimulus onset and offset, most transient oscillatory signals correlated with low-level brain patterns (V1). During stimulus presentation, sustained β (∼20 Hz) and γ (>60 Hz) power best correlated with V1, while oscillatory phase components correlated with IT representations. Surprisingly, this pattern of results did not always correspond to low-level or high-level DNN layer activity. In particular, sustained β band oscillatory power reflected high-level DNN layers, suggestive of a feed-back component. These results begin to bridge the gap between whole-brain oscillatory signals and object representations supported by local neuronal activations.

## Significance Statement

Brain oscillations are thought to play a key role in visual perception. We asked how oscillatory signals relate to visual object representations in localized brain regions, and how these representations evolve over time in terms of their complexity. We used representational similarity analysis (RSA) between MEG oscillations (considering both phase and amplitude) and (1) fMRI signals (to assess local activations along the cortical hierarchy), or (2) feedforward deep neural network (DNN) layers (to probe the complexity of visual representations). Our results reveal a complex picture, with the successive involvement of different oscillatory components (phase, amplitude) in different frequency bands and in different brain regions during visual object recognition.

## Introduction

Oscillatory neuronal activity is thought to underlie a variety of perceptual functions. Different frequency bands can carry information about different stimulus properties (e.g., whether the stimulus consists of coarse or fine object features; Smith et al., 2006; Romei et al., 2011), feedforward or feedback signals (van Kerkoerle et al., 2014; Bastos et al., 2015), or may reflect neuronal communication between different neuronal populations (Fries, 2005; Jensen and Mazaheri, 2010). Other studies have shown that different components of an oscillation (e.g., its power or phase) encode different types of sensory information (Smith et al., 2006).

Although neuronal oscillations are observed in different brain regions, and key theories hold that they reflect processing within, and communication between, brain regions (Fries, 2005; Jensen and Mazaheri, 2010), it has been difficult to pin down how large-scale brain oscillations are related to local patterns of neural activity, and how this relationship unfolds over time. This is because oscillatory activity is often studied with methods such as EEG or MEG, which have low spatial resolution. Although oscillatory signals with high spatial specificity can be recorded via local field potential recordings in humans or animals, these methods usually only target specific brain regions, and thus can only provide a partial view of oscillatory activity and its role in large-scale brain function. A direct link between large-scale oscillations and local neural activity is missing.

Here, we combine large-scale oscillatory signals recorded by MEG with local patterns of neural activity recorded with fMRI to bridge the gap between oscillatory components and different dimensions of object representation in the brain. Using representational similarity analysis (RSA; Kriegeskorte et al., 2008), we investigate the information carried by whole-brain oscillations obtained from MEG, and examine how this information evolves over time during an object recognition task.

We define three distinct dimensions of interest along which neural representations may unfold, and which are often conflated in the literature. First, we use the terms “early” and “late” to denote the temporal evolution of representations. Second, we differentiate between “low-level” and “high-level” stages of a processing hierarchy. Third, we consider the complexity of representations by distinguishing between “low-complexity” and “high-complexity” information (for example, higher-complexity might be characterized by additional nonlinear transformations of the input). In many information processing systems and in many typical experimental situations, these three dimensions are directly related to one another, as input information propagates over time through a succession of hierarchical stages, becoming more and more complex along the way. In such situations, the three dimensions of interest are in fact redundant and need not be further distinguished. But in systems with recurrence and feedback loops (like the brain), time, space, and information complexity are not always linearly related. For example, a lower hierarchical level (e.g., V1) can carry higher-complexity representations, later in time, as a result of feedback loops or lateral connections (Lamme and Roelfsema, 2000). In our terminology, such a representation would be classified as late in time, low-level in the hierarchy, yet high-complexity.

In this work, we consider two main hierarchical systems. We are interested in understanding information processing in the human brain, so we use V1 and IT fMRI brain representations, as done in a number of recent studies (Cichy et al., 2014; Khaligh-Razavi and Kriegeskorte, 2014). Representational similarity between MEG oscillations and this fMRI-based hierarchy can be interpreted in terms of early and late representations (based on the timing of the MEG oscillations), and in terms of low-level (V1) versus high-level (IT) hierarchical stages. To assess the complexity of representations independent of temporal evolution and cortical hierarchy of processing, we related our data to a second class of hierarchical systems: artificial feed-forward deep neural networks (DNNs), as done also in numerous recent studies (Cichy et al., 2016; Bankson et al., 2018; Hebart et al., 2018; Khaligh-Razavi et al., 2018; Kuzovkin et al., 2018). In these artificial networks, the hierarchical level (low-level vs high-level) is directly related to feature complexity (low-complexity vs high-complexity representations), because of the absence of feed-back or recurrent loops: the layer number directly reflects the number of nonlinear input transformations. For any MEG oscillatory signal, representational similarity with DNN activation patterns can thus inform us about representational complexity. In turn, any difference between DNN-based and brain-based RSA may be suggestive of feed-back or recurrent influences in the MEG oscillatory signals.

With this dual approach, we find an intricate picture of transient and sustained oscillatory signals that can be related to V1 and IT representations. Transient oscillatory components around stimulus onset and offset, as well as sustained β (∼20 Hz) and γ (>60 Hz) power components resemble V1 representations, while phase-dependent sustained activity correlates best with IT representations. However, when compared with DNNs, some low-level V1-like components actually correlate more with higher DNN layers, suggesting that stimulus representations in primary brain regions may already include high-complexity information, presumably as a result of feedback or top-down influences (Kar et al., 2019; Kietzmann et al., 2019).

In effect, our results narrow the gap between the description of neural dynamics in terms of whole-brain oscillatory signals and local neural activation patterns. Disentangling temporal evolution, hierarchical stage of processing and complexity of representations from each other, our approach allows for a more nuanced view on cortical information flow in human object processing.

## Materials and Methods

### Experimental paradigm and data acquisition

The data analyzed in this study was obtained from (Cichy et al., 2014), and detailed methods can be obtained from that paper.

**Figure 1.**
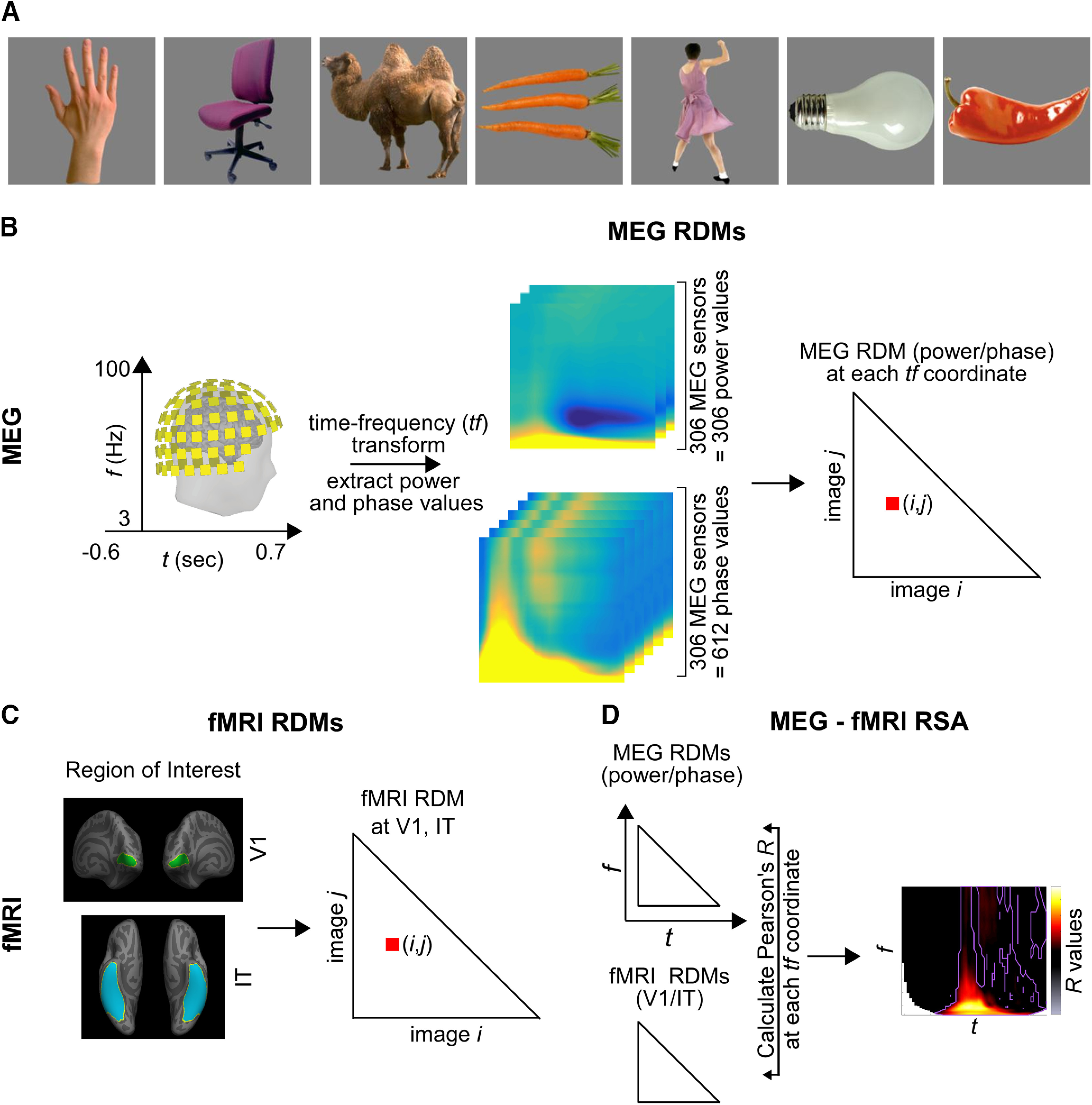
MEG-fMRI RSA analysis. ***A***, Examples from our 92-image set (***B***) MEG analysis and MEG RDMs. From the MEG signals, the complex TF transform was computed for each of the 306 MEG sensors. The amplitude and phase (separated into cosine and sine) values were extracted from the complex number at each TF coordinate, and a MEG RDM was constructed, reflecting the distance between oscillatory activation patterns for every pair of images (*i,j*; for details, see Materials and Methods). As a result, we obtained a power and phase MEG RDM at each TF coordinate for each participant. ***C***, fMRI RDMs were obtained from (Cichy et al., 2014). Two ROIs were defined: V1 and IT and one fMRI RDM was obtained for each ROI, and each participant, reflecting the distance between BOLD activation patterns for every pair of images (*i,j*). ***D***, RSA consists in comparing two (or more) RDMs. The MEG power or phase RDMs were compared with the fMRI RDMs (V1 or IT) by computing the partial Pearson’s *R*. This step was performed at each TF coordinate, resulting in an RSA map of R values at each TF coordinate, for each subject and ROI.

Fifteen human subjects of either sex performed separate MEG and fMRI sessions while they viewed a set of 92 images. The image set consisted of human and non-human faces and bodies, and artificial and natural everyday objects. The 92-image stimulus set was taken from the Kiani image set (Kiani et al., 2007), which consists of cutout objects on a gray background (Fig. 1*A*).

In the MEG sessions, each image was presented for 0.5 s followed by an interstimulus interval (ISI) of 1.2 or 1.5 s. Every three to five trials, a target paperclip object was presented, and subjects’ task was to press a button and blink whenever they detected this target image. Subjects performed two MEG sessions, of 2 h each. In each session they performed between 10 and 15 runs. Each image was presented twice in each run, in random order.

In each of two fMRI sessions, each image was presented for 0.5 s followed by an ISI of 2.5 or 5.5 s. Subjects’ task in the fMRI sessions was to press a button when they detected a color change in the fixation cross on 30 null trials, when no image was presented. Each image was presented once in each fMRI run, and subjects performed 10–14 runs in each session.

The MEG data were acquired from 306 channels (204 planar gradiometers, 102 magnetometers, Elekta Neuromag TRIUX, Elekta) at the Massachusetts Institute of Technology. The MRI experiment was conducted on a 3T Trio scanner (Siemens), with a 32-channel head coil. The structural images were acquired using a T1-weighted sequence (192 sagittal slices, FOV = 256 mm^2^, TR = 1900 ms, TE = 2.52 ms, flip angle = 9°). For the fMRI runs, 192 images were acquired for each participant (gradient-echo EPI sequence: TR = 2000 ms, TE = 32 ms, flip angle = 80°, FOV read = 192 mm, FOV phase = 100%, ascending acquisition gap = 10%, resolution = 2 mm, slices = 25).

### MEG analysis, preprocessing

MEG trials were extracted with a 600-ms baseline before stimulus onset until 1200 ms after stimulus onset. A total of 20–30 trials were obtained for each stimulus condition, session, and participant. Each image was considered as a different stimulus condition.

Data were analyzed using custom scripts in MATLAB (MathWorks) and FieldTrip (Oostenveld et al., 2011). Data were downsampled offline to 500 Hz. For each trial and sensor, we computed the complex time-frequency (TF) decomposition using multitapers. Parameters used were: 50 distinct frequencies increasing logarithmically from 3 to 100 Hz, over a time interval of –600 to 700 ms with respect to stimulus onset, in steps of 20 ms. The length of the sliding time window was chosen such that there were two full cycles per time window. The amount of smoothing increased with frequency (0.4 * frequency).

From the complex number at each TF coordinate, we extracted two measures for each sensor and each stimulus condition: the power and the phase of the oscillation. For each channel and stimulus condition, on each trial, the power was first expressed in decibels, and then averaged across trials to obtain one power value per stimulus condition. The phase of the oscillation was obtained by first normalizing each trial to make each trial’s vector in the complex domain of unit length, and then averaging across trials for each stimulus condition. The resultant average vector was then normalized to unit length, and the sine (real) and cosine (imaginary) components were extracted for each stimulus condition and each sensor.

### MEG analysis, multivariate analysis (Fig. 1*B*)

At each TF coordinate and for each stimulus condition, we next arranged the 306 power values from the 306 MEG sensors into a 306-dimensional vector representing the power pattern vector for that stimulus condition. Similarly, at each TF coordinate and for each stimulus condition we concatenated the 306 sine and 306 cosine values into a 612-dimensional phase pattern vector for that stimulus condition.

We next computed two representational dissimilarity matrices (RDMs): one for power and one for phase, at each TF point. For each pair of stimulus conditions, the power (phase) pattern vectors were correlated using the Pearson correlation measure, and the resulting 1-correlation value was assigned to a 92 × 92 power (phase) RDM, in which the rows and columns corresponded to the images being compared. This matrix is symmetric across the diagonal. This procedure results in one power (phase) RDM at each TF point.

### fMRI analysis (Fig. 1*C*)

The preprocessing steps for the fMRI data are described in detail in Cichy et al. (2014). For the multivariate analysis, two regions of interest (ROIs) were defined: V1 and IT. In each subject, for each ROI, voxel activation values were extracted for each stimulus condition, and the resulting values were arranged in a pattern vector for each stimulus condition. Then, in each ROI, for each pair of stimulus conditions, the corresponding pattern vectors were correlated using the Pearson correlation measure, and the resulting 1-correlation value was assigned to the 92 × 92 fMRI RDM. For further analysis, the fMRI RDMs were averaged across the 15 subjects, resulting in one RDM per ROI. The fMRI RDMs were provided by R. Cichy, D. Pantazis, and A. Oliva (Cichy et al., 2014).

### MEG-fMRI RSA (Fig. 1*D*)

RSA consists in comparing two (or more) RDMs. RSA between the MEG and fMRI RDMs was performed by computing the partial Pearson’s correlation between each MEG (phase or power) RDM with each fMRI RDM (V1 or IT), while partialling out any contribution from the other fMRI RDM (IT or V1). We chose to perform a partial correlation because the V1 and IT RDMs were positively correlated with each other (*r* ∼0.3); compared with a standard correlation, the partial correlation allowed us to isolate the unique correlation of each fMRI RDM with the MEG RDM, while discarding their joint contribution.

This procedure resulted in four RSA maps per subject (power/phase MEG RDMs × V1/IT fMRI RDMs). Each RSA map shows the *R* value between the MEG signals and the V1/IT activation patterns at each TF point (Figure 4). Significance of the RSA result was evaluated with a paired *t* test against 0, FDR corrected, α = 0.05. The timecourse of these effects is shown in Figure 5.

### Clustering analysis

**Figure 2.**
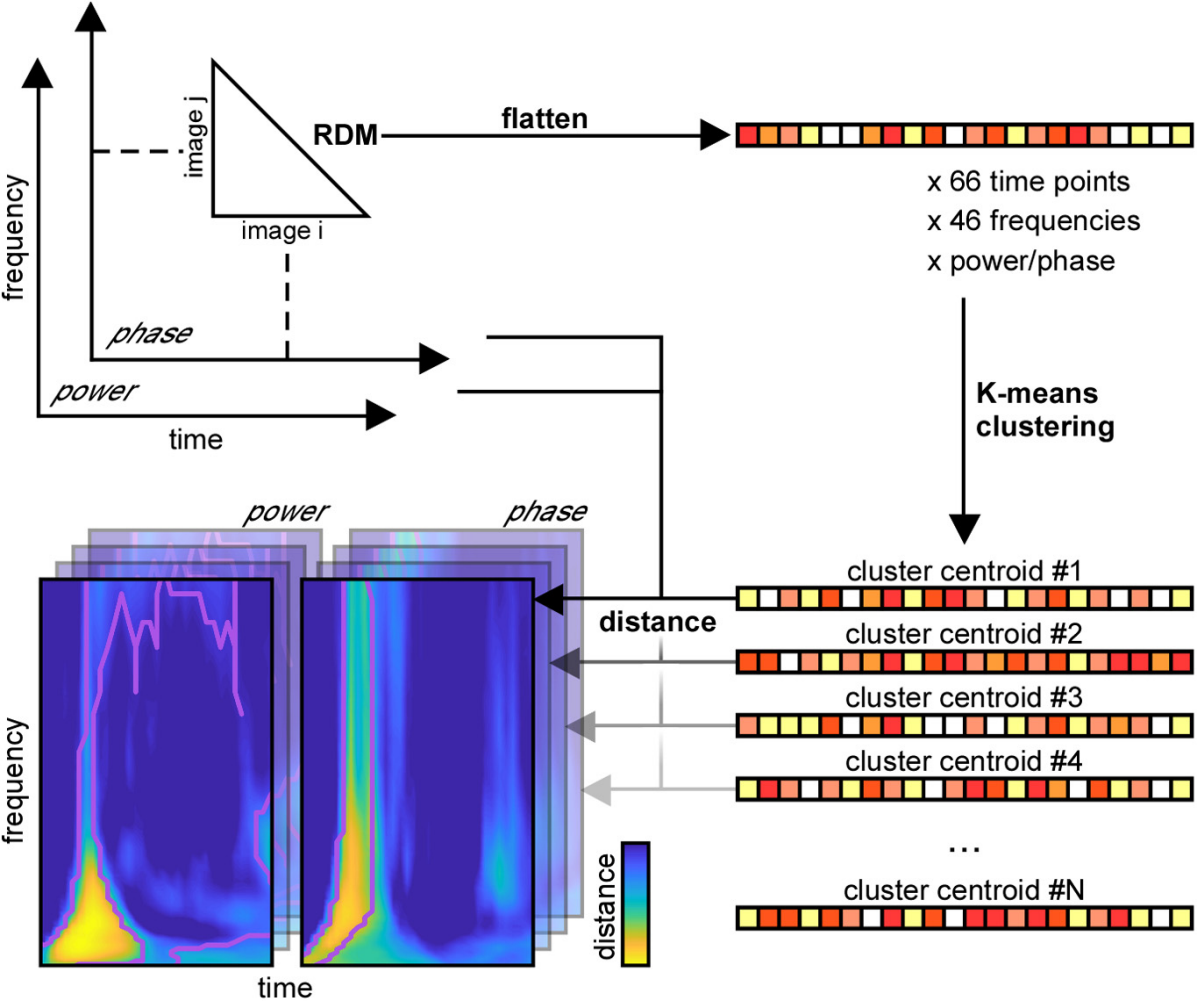
K-means clustering analysis procedure. Starting from the MEG RDM representations (top left, as described in Fig. 1*A*), we flatten each RDM data point into a vector. The entire set of vectors (across all TF coordinates and power/phase conditions) is entered into a K-means clustering algorithm (right), resulting in N clusters and their centroids. By measuring the distance of these centroids to all initial RDM data points, we obtain TF maps of “distance to centroid” (bottom left) that capture the main TF components (across both power and phase) of each cluster.

The MEG TF RDMs are heavily correlated with each other. To facilitate the interpretation of the information content of oscillatory signals, and to determine which features co-vary and which are independent, K-means clustering was performed on the MEG power and phase RDMs (Figure 2). Clustering was performed on the 4186-dimensional [(92 × 92–92)/2] RDMs across all (66) time points and (46) frequency points, combining the power and phase signals (resulting in 46 × 66 × 2 = 6072 data points to cluster in a 4186-dimensional space). K-means was implemented with the MATLAB function kmeans, with the correlational distance measure, five replicates, and the number of clusters going from 1 to 20. The optimal number of clusters was then determined with the elbow criterion defined as the point just before the local maximum of the second derivative of the residual sum of squares (corresponding to the point at which adding another cluster would only provide a marginal gain in variance explained). With this method, the first elbow occurred at *k *=* *7 clusters.

The chosen clusters could be visualized by plotting the correlation distance (in the 4186-dimensional RDM space) between the cluster’s centroid and every TF point, for both power and phase signals, resulting in two TF maps of “distance to cluster centroid” for each cluster (Figs. 6, 7).

RSA (using partial Pearson’s correlation) was performed between each cluster’s centroid and each of the fMRI RDMs (see below). For RSA with fMRI, this procedure resulted in two RSA values *R_V1_* and *R_IT_* (one each for V1 and IT). Since each cluster centroid could correspond to both V1 and IT to different degrees, the “cortical level” *L* of the cluster was positioned somewhere between V1/low-level and IT/high-level using the following equation:

(1) L=σ((RIT−RV1)(RIT + RV1)),

where σ denotes the sigmoid function. The measure *L* could vary between 0 (when the cluster’s representational content was perfectly similar to V1) and 1 (when it was perfectly similar to IT).

Significance of RSA between the cluster centroids and the fMRI RDMs was computed with a surrogate test. On each iteration, the cluster centroid RDM was randomly shuffled and the partial correlation was computed between this shuffled RDM and the true RDM. This procedure was repeated for 10^5^ iterations, and the proportion of iterations on which the shuffled RSA values were higher than the true RSA values was counted.

### DNN RDMs

**Figure 3.**
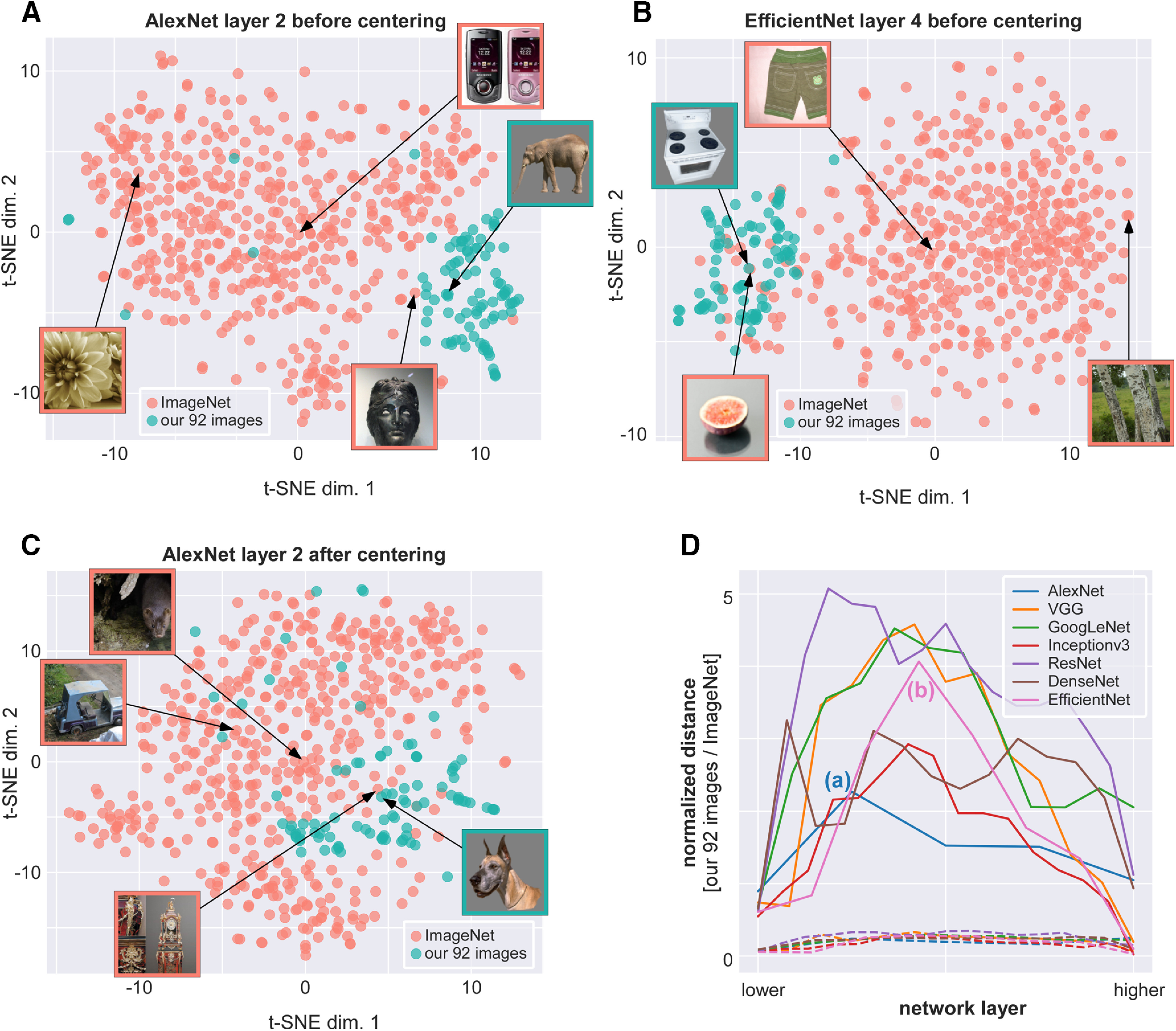
***A***, ***B***, t-distributed stochastic neighbor embedding (t-SNE) visualizations of 500 ImageNet samples and the 92-image stimulus-set used in this study, across representative layers of two networks (***A***, AlexNet; ***B***, EfficientNet). To obtain these visualizations, the feature values of all images were subjected to a principal component analysis (PCA) of which only the first 100 dimensions were retained (so as to limit computational demands); then, t-SNE was applied, as implemented in the scikit-learn Python library, with parameters: [perplexity = 30, n_iter = 1000, learning_rate = 1.0, min_grad_norm = 0]. The DNNs used in this study had been trained on images from ImageNet, which consists of millions of photographs of one or more objects in natural backgrounds. In contrast, our 92-image stimulus set consists of cut-out images on a gray background. The DNNs learn optimal representations for the training images from ImageNet, i.e., different images from different categories are mapped to different regions of the representation space, and the whole space tends to be equally occupied by the training samples. However, as the t-SNE visualizations show, our 92 images are all projected into a remote corner of this space, meaning that the RDM distances between the 92 images are confounded by the mean vector (the pairwise Pearson distance depends more on the alignment with the mean vector, and less on the true physical distance between points). Inset images show the most stereotypical image of our 92-stimulus set (highlighted in green), the closest image from the ImageNet set (characterized, as expected, by an empty gray background), as well as one ImageNet sample near the space origin, and one on the opposite side of the feature space. To circumvent this problem, we used a re-centering approach as described in Materials and Methods. ***C***, The same layer of AlexNet as shown in ***A***, after re-centering. The 92-stimulus set is now closer to the center of the feature space. ***D***, Systematic measurement of the distance between the centroid of our 92-stimulus set and the space origin (normalized by the SD across our 92 images), for each layer of each DNN. The two DNN layers depicted in ***A***, ***B*** are labeled (a) and (b) on the corresponding curves. As a baseline, the dashed lines reflect the same distance measure, applied to the 500 ImageNet samples.

The MEG phase/power representations were also compared with representations in seven DNNs (so as to ensure that conclusions were not dependent on one specific network architecture): AlexNet (Krizhevksy et al., 2012), VGG16 (Simonyan and Zisserman, 2015), GoogleNet (Szegedy et al., 2015), InceptionV3 (Szegedy et al., 2017), ResNet50 (He et al., 2016), DenseNet121 (Huang et al., 2017), and EfficientNetB3 (Tan and Le, 2019), processing the same 92 images as in our MEG and fMRI data. However, in contrast to our 92-image stimulus set, which consisted of cutout objects on a gray background, the DNNs had been trained on images from ImageNet (millions of photographs with one or more objects in natural backgrounds). The networks had thus learned optimal representations for their training set, but in this representation space our 92 images tended to cluster into a remote “corner” (Fig. 3), with low dissimilarity (1- Pearson’s *R*) values between images, and a resulting RDM of poor quality. To retrieve meaningful distances between the representations of the 92 images, we first performed a centering procedure: we centered the activation of each layer of each DNN by subtracting the mean activation of an independent set of 368 images from the Kiani image set. This independent image set consisted of four images from each of the categories in our 92-image set. Importantly, because the image set used for centering did not include any of the 92 images from our study, there was no circularity in the centering operation, nor any leakage of information between the representations of our 92 images. This centering procedure contributed to minimize the potential problems arising from differences between our dataset and the standard ImageNet dataset, but it did not completely alleviate these differences, as can be seen in Figure 3*C*.

RDMs were constructed for several convolutional layers of each network based on the layer activation values. There were five layers for AlexNet, 13 for VGG16, 12 for GoogleNet, 16 for InceptionV3, 17 for ResNet50 (hereafter referred to as ResNet), 14 for DenseNet121 (hereafter DenseNet), and eight for EfficientNetB3 (hereafter EfficientNet). These layers were chosen so as to span the entire network hierarchy, without making the analysis computationally intractable (as some networks can contain >200 layers to choose from). RSA was then performed (with the Spearman correlation) between these RDMs and the centroid of each cluster (see above for details of the clustering analysis). The layer with maximum RSA, normalized by the number of layers for this DNN, was taken to reflect the information content of this cluster between 0 (in the terminology defined in the Introduction, low-complexity corresponding to the DNN’s first layer) and 1 (high-complexity, corresponding to the DNN’s last layer), and finally averaged across the seven DNNs.

### Code accessibility

Custom code can be made available on request.

## Results

Fifteen participants viewed the same set of 92 images while fMRI and MEG data were recorded (in separate sessions). The image set consisted of human and non-human bodies and faces, and artificial and natural stimuli. Each stimulus was presented for 0.5 s, followed by a 1.2- or 1.5-s baseline period.

To assess oscillatory components, we extracted stimulus-related activity from –600 to 1200 ms relative to stimulus onset from the MEG data. For each trial, and each sensor a TF decomposition was performed, and a power and phase value extracted at each time and frequency point. These values were used to compute RDMs at each TF point, separately for power and phase (Materials and Methods; Fig. 1*A*). Each element in the MEG RDMs indicates how distinct the corresponding images are in the MEG power or phase spaces, and the entire MEG RDM is a summary of how the 92-image stimulus set is represented in the MEG oscillatory power or phase at each TF point.

To assess local patterns of neural activity, we generated fMRI RDMs by performing comparisons between the local BOLD activation patterns of pairs of images in V1 and IT (Cichy et al., 2014). Two fMRI RDMs were obtained (Fig. 1*B*), one for V1 and one for IT. The fMRI RDMs are a measure of the representation of the image set in the voxel space of V1 and IT local neural activity.

### Bridging the space, time, and frequency gap in object recognition

**Figure 4.**
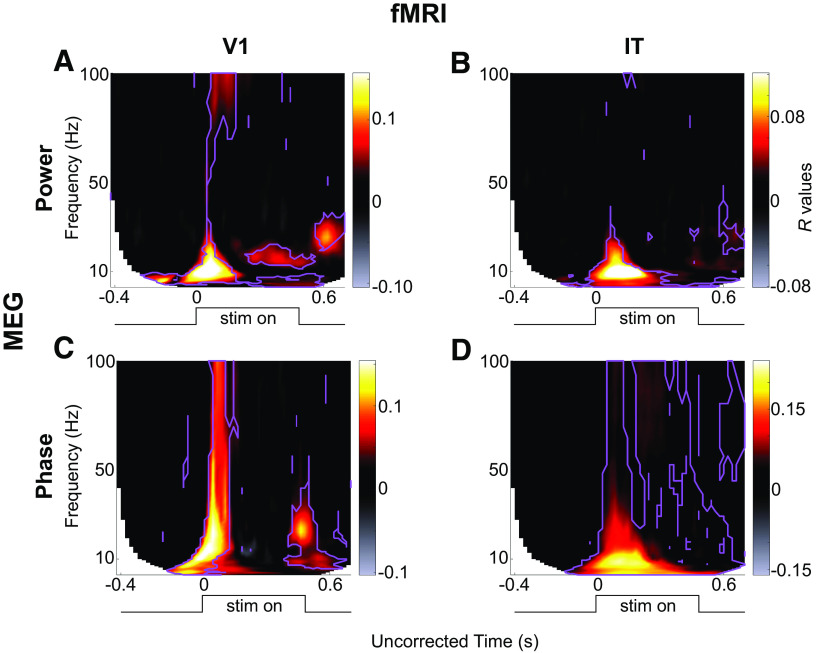
Results of the 2 × 2 RSA comparisons (MEG power/phase × fMRI V1/IT), averaged over all subjects. (***A***) MEG-Power x fMRI V1, (***B***) MEG-Power x fMRI-IT, (***C***) MEG-Phase x fMRI-V1, (***D***) MEG-Phase x fMRI-IT. The purple contours mark those regions in the maps that are significantly different from zero (paired *t* test against 0 across *N *=* *15 subjects, FDR correction, α = 0.05). Note that the absolute latencies are not directly comparable across frequencies, because of different smoothing windows applied at the different frequencies when performing the TF transform (hence, the *x*-axis is labeled as uncorrected time).

How similar is the oscillatory representation of the images to their representation in each brain region? The MEG RDMs (power and/or phase) at each TF point represent the stimulus set in a large-scale brain oscillatory activity space, while the fMRI RDMs represent the same image set via BOLD activity in a local population of neurons in two brain regions (V1 or IT). We evaluated the similarity of representations in the TF domain with those in the fMRI activation patterns by computing the partial Pearson’s correlation between the MEG RDMs (phase or power) with the fMRI RDMs (V1/IT), at each TF point (Fig. 1*C*). This analysis resulted in four TF maps of *R* values (or RSA maps), which provide the unique correspondence between whole-brain oscillations and local patterns of neural activity in V1 and IT, at each TF point (Fig. 4). With these maps we can ask whether and when stimulus information contained in oscillatory phase or power at each TF point resembles BOLD activations in a given brain region (V1/IT), and potentially, which region it resembles more. The advantage of measuring partial correlation (instead of a standard correlation) is to discard the (potentially large) portion of the variance in oscillatory representations that is explained equally well by V1 or IT BOLD representations, owing to the fact that V1 and IT signals already share similarities. This way, we concentrate on the part of oscillatory representations that is uniquely explained by each brain ROI.

**Figure 5.**
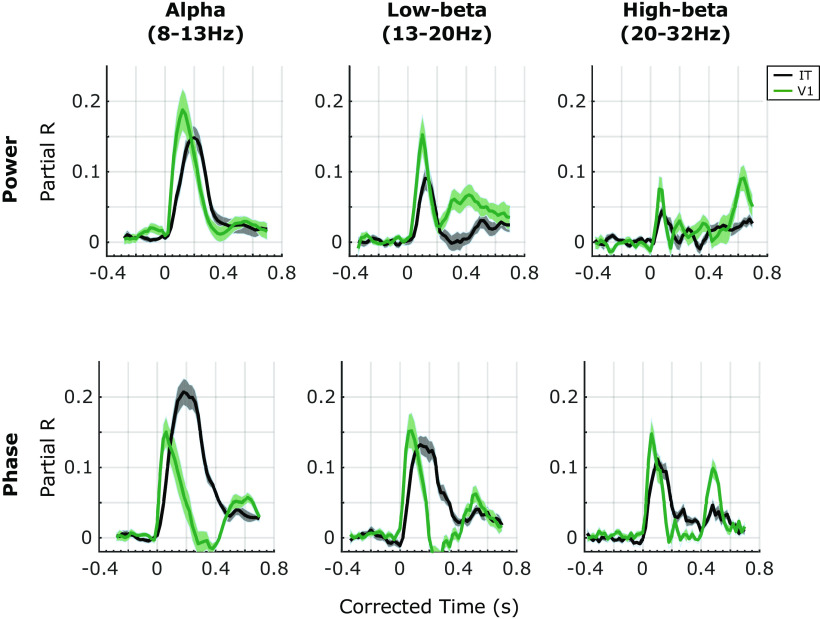
Profile of the results of the RSA with V1 (green lines) and IT (black lines) in oscillatory power (top row) and oscillatory phase (bottom row) in different frequency bands. To examine the RSA maps in more detail, we extracted their time courses in different traditional frequency bands: α (8–13 Hz), low-β (13–20 Hz), and high-β (20–32 Hz). In each of these frequency bands, we computed the average *R* values. Since the TF decomposition induces temporal smearing, and the amount of smearing differs for different frequencies, to interpret the latencies of the representational similarities, we corrected for this smearing effect. Specifically, to avoid underestimating the onset latencies, we corrected time by adding half the wavelet window duration at each frequency. Note that the same correction was applied to the two curves compared in each plot. Solid lines are the means across subjects, and the shaded areas correspond to the SEM across subjects.

Our results show that different oscillatory components map to different brain regions at different moments in time. Overall, the absolute maximum of representational similarity with brain area V1 occurred in the α band ∼120 ms after stimulus for oscillatory power, whereas the absolute maximum related to area IT occurred for θ and α phase ∼200–300 ms. More generally, a strong increase in representational similarity was observed shortly after stimulus onset in all four maps. The frequency, latency and duration of these similarity effects depended however on the exact oscillatory signal (power, phase) and brain region (V1, IT). In terms of MEG power (Fig. 4*A*,*B*), the latencies (see also Fig. 5) respected the hierarchical order of visual processing (Nowak and Bullier, 1998) with an increase in representational similarity in the lower (<20 Hz) frequency bands occurring around the evoked response first for RSA with V1, and ∼20–30 ms later for RSA with IT (paired *t* test against 0, FDR corrected, α = 0.05). This latency difference is similar to that reported in Cichy et al. (2014), where the peak correspondence between the average MEG signal and V1 activity occurs ∼30 ms before the peak with IT activity. The onset response in the V1 RSA map also consisted of high γ frequencies (>70 Hz), whereas this high-γ activity was not observed in the IT RSA map. This is compatible with recent findings showing that γ oscillations in early visual cortex are particularly prominent for certain stimuli, yet can be entirely absent for others (Hermes et al., 2015). A sustained low-β (20 Hz, 200–500 ms) and an offset high-β (30 Hz, ∼600 ms) response also corresponded to V1 representations, although neither of these effects were observed in the IT RSA map (see also Fig. 5). In terms of stimulus representations in the MEG oscillatory phase (Fig. 4*C*,*D*), after an initial broadband (3–100 Hz) transient peak at stimulus onset corresponding to V1 representations, stimulus information carried by sustained oscillatory phase resembled IT representations in the low (<20 Hz) and high frequency (60 Hz) bands, and this resemblance persisted until the end of the trial. Phase representations corresponding to V1 patterns were observed again around stimulus offset, at α (∼10 Hz) and β (20–30 Hz) frequencies (see also Fig. 5), in line with the known involvement of V1 neurons in OFF responses (Jansen et al., 2019).

**Figure 6.**
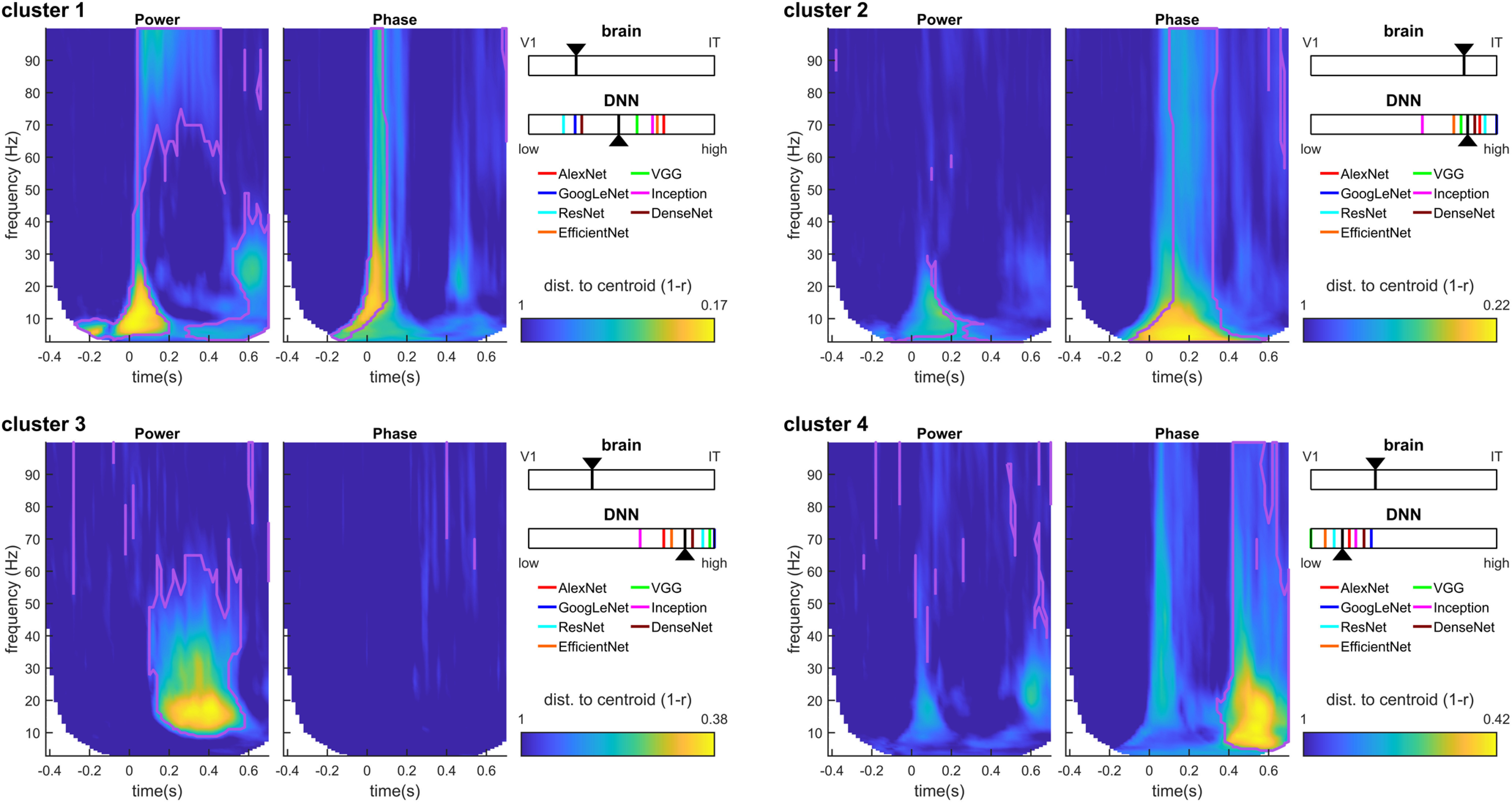
Clustering analysis. K-means clustering was performed on the MEG power and phase RDMs. Each TF plot shows the distance of each RDM from the centroid of the corresponding cluster. The purple lines correspond to the cluster boundaries as returned by the k-means algorithm, indicating that all points within the purple lines are assigned to this specific cluster based on their distance to the different cluster centroids. The distance to centroid (color scale) reflects how “stereotypical” each RDM is for the corresponding cluster (i.e., how close to the cluster centroid), a continuous scale that complements the discrete cluster assignment. For example, although cluster 3 simultaneously encompasses oscillatory power across many frequencies from 10 to 65 Hz, we can see that low-β frequencies (13–20 Hz) are the most stereotypical for this cluster. The insets show the relative degree of RSA between the cluster centroid and V1/IT (top), or the cluster centroid and the DNN layer hierarchy (bottom). For the DNNs, the layer with maximum RSA, normalized by the number of layers in the DNN hierarchy, and averaged across the seven DNN types (colored ticks), was taken as the layer that corresponded to each cluster centroid (black arrowhead).

**Figure 7.**
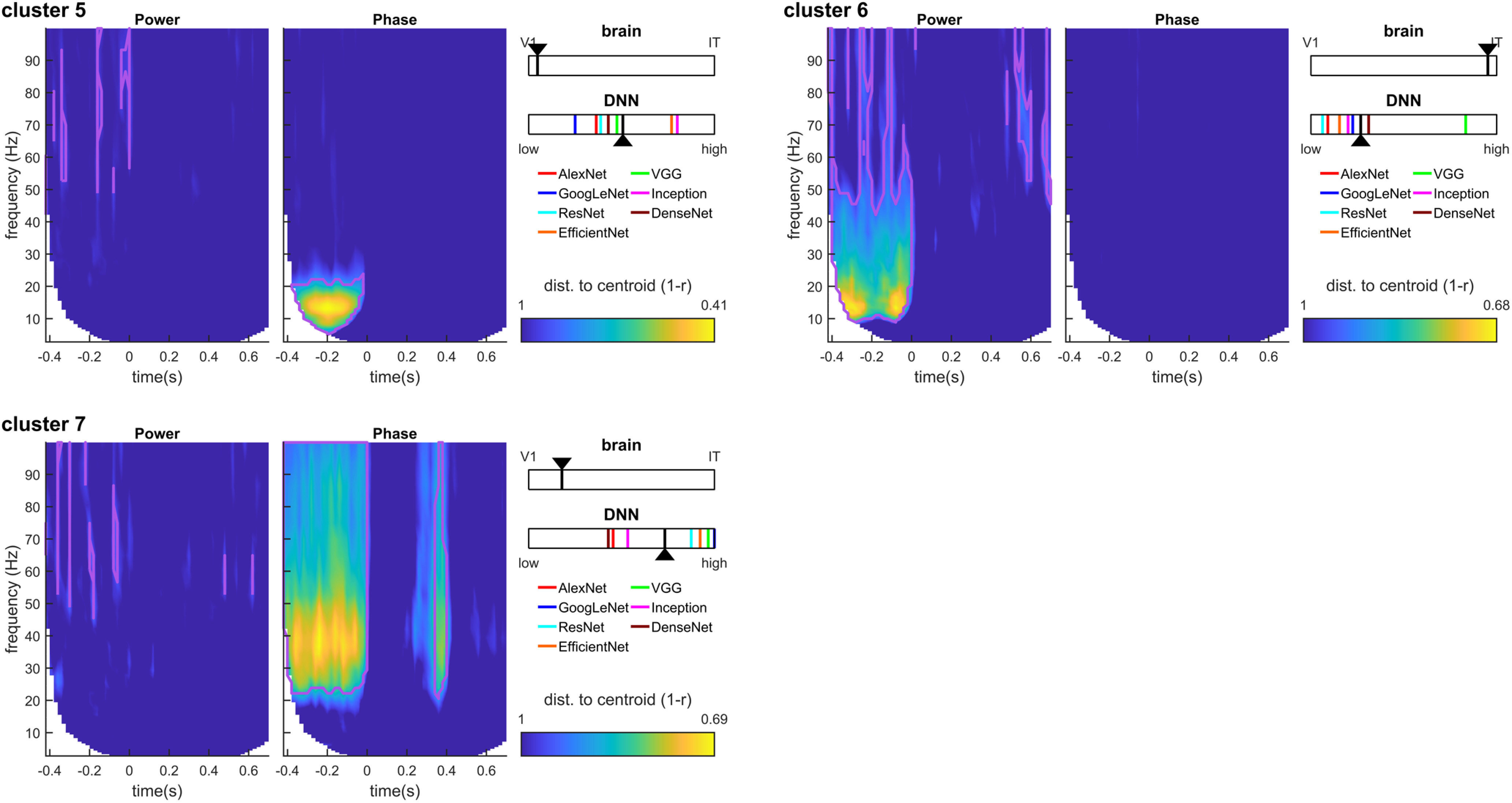
Clustering results for clusters 5–7. We identify these clusters as noise components because (1) their distance to the cluster centroid is typically higher than for other clusters, and (2) they mainly map onto prestimulus oscillatory activity. Prestimulus oscillations, while accounting for a sizeable portion of the (notoriously noisy) MEG signal variance, cannot possibly encode the identity of a stimulus that has not been presented yet. Prestimulus α is a well-studied oscillatory component reflecting the attention state of the observer, and whose phase is known to modulate the subsequent ERP amplitudes and latencies; as such, it is not surprising that the phase of this oscillatory component would induce a separate cluster of RDM patterns (cluster 5). Similarly, prestimulus α-β power (cluster 6) and γ phase (cluster 7) could reflect preparatory attention or motor signals (including muscular artifacts) not related to stimulus identity. Notations as in Figure 6. Note that for consistency with the previous figure, we continue to report the V1-IT RSA scaling value in the insets; however, the corresponding correlation values were systematically lower for these clusters, and should thus be interpreted with caution (V1 partial correlations for clusters 5–7: [0.04, 0.00, 0.08], IT partial correlations: [0.00, 0.04, 0.03]). In comparison, V1 partial correlations for clusters 1–4 ranged from 0.14 to 0.43, and IT partial correlations from 0.12 to 0.44. Similarly, we also report the DNN layer for which the correlation to the cluster centroid was maximal; however, this maximal correlation was consistently lower than in the previous figure, as expected for a prestimulus component (peak correlations averaged across DNNs for clusters 5–7: [0.02, 0.03, 0.16], compared with values ranging from 0.24 to 0.45 for clusters 1–4).

These results thus suggest that different oscillatory components correspond to different brain regions at different TF points. However, since the RDMs in the TF space are heavily correlated with each other, it is difficult to ascertain from this analysis which power/phase features co-vary, and which effects occur independently. To better interpret the results shown in Figure 4, we turned to a clustering analysis. The clustering analysis allowed us to reduce the dimensionality of the dataspace and to determine which oscillatory signals occurred jointly, and which are independent. We performed k-means clustering jointly on the power and phase RDMs. That is, each RDM (one for each time point, frequency, and phase/power signal), was considered as an input data point for the clustering analysis, which returned the corresponding cluster index assigned to each point (see Fig. 2). The results of the clustering analysis for *k *=* *7 clusters (the optimal number of clusters for our dataset) are shown in Figure 6 (see also Fig. 7). The first cluster (ranked by smallest average distance from cluster centroid) corresponded to early broadband (0–100 Hz) phase and power RDMs, followed by sustained γ power (>60 Hz), and β power (20–30 Hz) at stimulus offset. The second cluster corresponded to broadband (0–100 Hz) and sustained (0.1–0. 4s) phase effects after stimulus onset, without any noticeable power effects. The third cluster consisted primarily of sustained (0.1–0.6 s) β (10–30 Hz) and low-γ (<60 Hz) power, without any noticeable phase effects. The fourth cluster reflected broadband phase effects (0–100 Hz) at stimulus offset (without associated power effects). The last three clusters (5–7) all displayed prestimulus effects in α-β power, or α or γ phase, characteristic of spontaneous, stimulus-unrelated activity that we did not investigate further (Fig. 7). The clustering analysis performed on the MEG RDMs thus identified four main clusters of power and phase oscillatory components that occurred at different time points and in different frequency bands after stimulus onset.

How do the oscillatory representations in each cluster, and their different time and frequency profiles relate to local processing in V1 and IT as measured by fMRI representations? To address this question, we performed RSA between the cluster centroids and the V1 and IT RDMs. The cluster centroids correlated to different degrees with both V1 and IT (all partial *R* values between 0.12 and 0.49; all significant at *p *<* *1e-5 with a surrogate test; see Materials and Methods). To directly contrast the representational similarity of each brain area (V1, IT) to the cluster centroid, we combined the two RSA partial *R* values into a single scale (see Materials and Methods, Eq. 1). According to this scaling (Fig. 6, insets), the transient broadband phase and power effect with sustained γ power in cluster 1 corresponded best with V1 representations (i.e., low-level). Conversely, the broadband sustained phase effects of cluster 2 corresponded best to IT representations (high-level). The other two clusters (sustained β-γ power in cluster 3, broadband offset-transient phase in cluster 4) had more balanced similarity to both V1 and IT, with a slight inclination toward V1. Thus, transient oscillatory components occurring around stimulus onset correspond more closely to V1 representations, whereas the more sustained components could be either more IT-like, or less localized depending on the frequency of the oscillations. These results thus suggest a complex link between oscillatory representations and local processing in V1 or IT. To try to clarify these relationships we next turned to using DNNs as a template for object representations.

### Assessing representational complexity with DNNs

The fMRI RDMs are a representation of the image set in the multi-voxel space of V1 and IT. However, these fMRI representations are static because the fMRI BOLD signal used to construct the RDMs was measured over a period of several seconds. Neuronal activity in these regions, on the other hand, is known to evolve over fairly rapid timescales, on the order of hundreds of milliseconds as a result of feedback and top-down signals (Roelfsema et al., 1998; Lamme and Roelfsema, 2000). The fMRI RDMs are thus limited representations of the image set, potentially mixing low-complexity and high-complexity brain activity from different moments in each trial. Therefore, while it is tempting to interpret the oscillatory signals composing cluster 1 as low-complexity, because they are more V1-like, and those forming cluster 2 as high-complexity (more IT-like), such a conclusion would be premature as it ignores the dynamics of neural responses within and across brain regions, and how these neural responses evolve over different timescales. To obtain a complementary picture of low and high-level object representations, we considered the representations of our image set in different layers of feed-forward DNNs pretrained on a large dataset of natural images. To ensure the generality of our results we assessed seven different DNNs: AlexNet (Krizhevksy et al., 2012), VGG16 (Simonyan and Zisserman, 2015), GoogleNet (Szegedy et al., 2015), InceptionV3 (Szegedy et al., 2017), ResNet (He et al., 2016), DenseNet (Huang et al., 2017), and EfficientNet (Tan and Le, 2019). Activity in each layer of these DNNs is not influenced by top-down or recurrent connections, and consequently represents a truly hierarchical evolution in the complexity of image representations, from low to high complexity. Indeed, several studies have suggested that DNN representations approximate the feed-forward cascade of the visual processing hierarchy in the brain (Khaligh-Razavi and Kriegeskorte, 2014; Cichy et al., 2016). Performing RSA between MEG oscillatory RDMs and DNN layer RDMs should thus reveal which features of the MEG oscillatory representations correspond to low-complexity vs high-complexity object representations.

An RDM was obtained for several representative convolutional layers of the seven DNNs. RSA was then performed between the cluster centroids of the MEG RDMs and the DNN RDMs. For each cluster and DNN, the layer with maximum RSA was determined, and scaled between 0 (lowest layer, low-complexity information) and 1 (highest layer, high-complexity information) based on the number of layers in the DNN hierarchy. Despite notable differences between the seven DNNs, the analysis revealed that clusters 2 and 3 mapped best to higher DNN layers, cluster 1 to intermediate layers, and only cluster four had similarity to lower layers. This is in stark contrast with the results of fMRI RSA, which had ranked clusters 2, 4, 3, and 1 in order of decreasing complexity. The most striking difference is obtained for cluster 3 (sustained β-γ power): a high-complexity representation according to DNNs, but closer to V1 than to IT according to fMRI. Based on the logic above, this cluster is likely to reflect feed-back signals that carry high-complexity object information (visible in high DNN layers) down to lower brain regions (visible in V1 BOLD signals).

## Discussion

**Figure 8.**
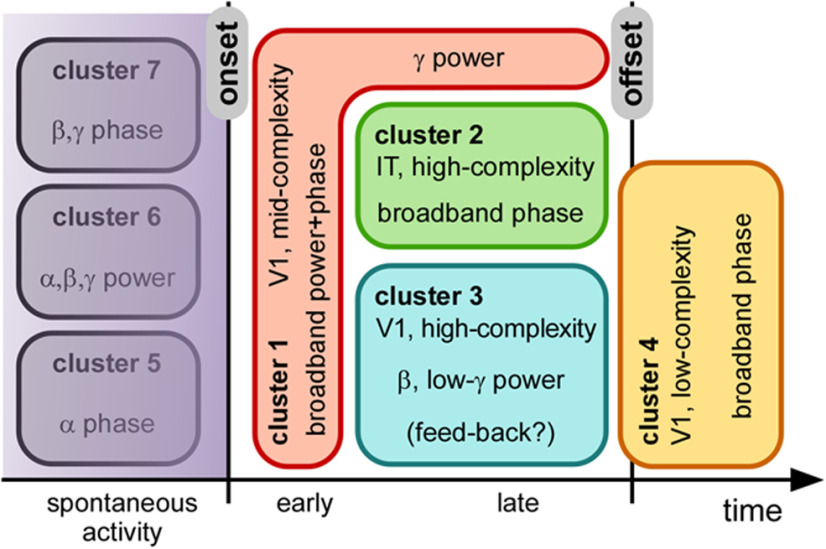
Illustrative summary. The different clusters identified are plotted schematically as a function of time, and their main oscillatory characteristics (frequency band, power/phase) are indicated, together with the corresponding brain region (V1/IT) and the corresponding DNN layer (low/mid/high-complexity).

Our results (summarized in Fig. 8) show that MEG oscillatory components at different frequencies carry stimulus-related information at specific times, which can be linked, via RSA, to stimulus representations in different brain regions (V1, IT), and with different representational complexity (as measured by DNNs). Importantly, the representational dynamics of brain oscillations can be very differently expressed by power versus phase signals. At stimulus onset and offset, broadband phase transients (possibly related to fluctuations in evoked potential latencies) carry mainly low-complexity or intermediate-complexity information (Fig. 6, clusters 1 and 4). However, during stimulus presentation, sustained phase information is visible across all frequencies, and consistently maps to high-level and high-complexity representations (IT and high DNN layers, cluster 2). Oscillatory power components (clusters 1 and 3) tend to correlate with both V1 and IT fMRI representations (with an inclination toward V1); however, onset-transient low-frequency (<20 Hz) power together with sustained high-frequency (>60 Hz) power (i.e., cluster 1) correspond best to intermediate DNN layers, whereas sustained β-γ power (20–60 Hz) clearly maps to the highest DNN layers (cluster 3).

It is important to note that some of the TF components revealed here could be specific to the conditions of our experiment. For example, brain oscillations are often modulated by the subjective state, the participant’s attention or the task instructions. As such, it is likely that different oscillatory patterns would be obtained for tasks involving active behavior rather than passive viewing of the images. Similarly, spurious muscular activity (including ocular saccades or microsaccades) could be an important contribution to the observed TF components (Yuval-Greenberg et al., 2008), as long as this activity would systematically differ between the different stimulus classes.

Our findings complement those reported in an earlier study that contrasted oscillatory power measured from intracranial electrodes with object representations across various layers of AlexNet (Kuzovkin et al., 2018). These authors linked γ power in lower visual areas to object content in lower DNN layers, while γ power in higher visual areas (as well as θ-band activity) was related to higher DNN layers. Possible differences with our own observations could be explained by the spatial scale of the electro-magnetic signals recorded (more localized in intracranial electrodes, more widespread in MEG), our consideration of phase in addition to power components, our use of 6 other DNNs in addition to AlexNet, or by more specific aspects of our analysis pipeline such as the DNN layer centering procedure (Fig. 3) or the K-means clustering (Fig. 2).

In our study, we found no simple mapping between low/high-level (or low/high-complexity) representations and oscillatory components (power/phase) or frequency. Both low-frequency (θ, α) and high-frequency (β, γ) oscillatory signals can carry either low- or high-level/complexity representations at different times (e.g., clusters 2 vs 4). Similarly, both phase and power signals can carry either low or high-level representations (e.g., clusters 1 vs 3). The picture that emerges is a rather intricate one, in which successive interactions between different oscillatory components in different brain regions and at different frequencies reflect the different stages of neural processing involved in object recognition.

How can the representational content of a given oscillatory component (phase or power) be interpreted in functional terms, at the level of neural populations and their interactions? For example, a sustained phase component (such as the broadband component in cluster 2) means that for some extended period of time (here, roughly between 150 and 350 ms), the exact phase of oscillatory signals (here, across multiple frequency bands, from δ to high γ) will systematically vary with the stimulus identity. Note that this is not about increased phase locking, but about the phase values themselves, and their differences between images. Such systematic phase differences between stimulus classes could arise if the underlying oscillatory processes come into play with different delays, e.g., as a result of information routed through slightly distinct circuits. As for oscillatory power, the differences (for example, the sustained β power differences summarized in Cluster 3) would imply that some image classes tend to result in higher amplitudes and others in weaker amplitudes. This could arise, for example, in a scenario where the oscillation is selectively triggered by certain images (those that the neural population is selective to, e.g., animate vs inanimate, natural vs man-made, etc.).

Our results highlight the importance of complementing MEG-fMRI RSA with another measure of representational content such as feed-forward DNNs (Cichy et al., 2016; Bankson et al., 2018; Hebart et al., 2018; Khaligh-Razavi et al., 2018). fMRI BOLD signals are often analyzed such that they reflect a single static representation. Thus, they cannot distinguish dynamics in local patterns as, for example, early feedforward and later feedback activity. By design, feedforward DNN layers cannot be dynamically influenced by feedback signals, and could be considered to provide a template for low-complexity versus high-complexity representations during the different stages of image processing. Perhaps the best illustration of this notion stems from the discrepancy between fMRI and DNN RSA for MEG cluster 3, which suggests that sustained β-γ power during stimulus presentation could reflect feedback signals: best corresponding to V1 fMRI activity (low-level), but higher DNN layers (high-complexity). Without this additional information (e.g., looking at Fig. 4*A* alone), one might have interpreted sustained β power as a strictly low-level signal. The observed distinction between sustained power effects at lower frequencies (β and low-γ, cluster 3) versus higher frequencies (high-γ, cluster 1) is consistent with a large number of recent studies that reported a functional distinction between γ-band and β-band signals, respectively supporting feed-forward and feedback communication (Fontolan et al., 2014; van Kerkoerle et al., 2014; Bastos et al., 2015; Michalareas et al., 2016). Future work could attempt to separate feedforward from feedback signals (e.g., with backward masking and/or layer-specific fMRI), to confirm the differential contribution of γ-band and β-band oscillatory frequencies to feedforward versus feedback object representations, as determined with RSA.

In addition to their involvement in the transmission of feedforward and feedback signals, several studies have shown that different oscillatory signals can carry distinct information about stimulus properties (Smith et al., 2006; Romei et al., 2011; Schyns et al., 2011; Mauro et al., 2015). Here, we considered whether oscillatory components in different frequencies correspond to lower-complexity or higher-complexity stimulus processing stages. Our results suggest that most oscillatory brain activity, at least at the broad spatial scale that is measured with MEG, reflects already advanced stimulus processing in object detection tasks. This result can be seen in Figure 6, where most oscillatory components are more related to higher-level DNN layer representations, with the exception of the offset-transient (cluster 4). Indeed, one might have expected that stimulus representations at both stimulus onset and offset are more reflective of transient low-level and low-complexity processing. However, while both onset and offset signals (clusters 1 and 4) are better matched to V1 than IT (low-level; see also Fig. 4*C*,*D*), in terms of DNN activations the offset-transient (cluster 4) appears to be of much lower-complexity and the onset-transient of higher-complexity (cluster 1). A tentative explanation could be that the continued presence of the stimulus after the onset-transient supports a rapid refinement of object representations, which would not be the case for the offset-transient (because the stimulus is absent from the retina). Indeed, it is remarkable that, aside from this offset-transient broadband phase activity (cluster 4), no other oscillatory signal was found to reflect low-level DNN layers (i.e., low-complexity information).

One possible explanation for the relative dearth of oscillatory components reflecting low-level DNN layers could be that neural oscillations are a circuit-level property, rather than a single-neuron property; this could provide a better match for high-level DNN layers that pool across large numbers of inputs. In any case, such a bias, if it exists, would only be relative, as we did find at least one oscillatory component (related to cluster 4) that better matched low-level DNNs.

In conclusion, our results help characterize the representational content of oscillatory signals during visual object perception. By separately considering hierarchical level (V1/IT) and representational complexity (based on DNNs), we narrow the gap between whole-brain oscillations and visual object representations supported by local neural activation patterns.

## Synthesis

Reviewing Editor: Nicholas J. Priebe, University of Texas at Austin Decisions are customarily a result of the Reviewing Editor and the peer reviewers coming together and discussing their recommendations until a consensus is reached. When revisions are invited, a fact-based synthesis statement explaining their decision and outlining what is needed to prepare a revision will be listed below. The following reviewer(s) agreed to reveal their identity: Chou Hung.

SYNOPSIS

Two reviewers evaluated the manuscript “Representational content of oscillatory brain activity during object recognition” and both highlighted the important role of the manuscript in linking oscillatory activity and object identification. Both reviewers noted that the present manuscript raises important questions about the level of processing that occurs in V1 and IT. But there was also a major concern, in review and in consultation, that in its present form the manuscript does not provide theretical predictions to link these observations to the circuitry for object identification. The impact of the manuscript would be substantialy increased if the complexity of the signals were placed into a theoretical framework. In addition, both reviewers provided detailed suggestions which you should find useful in revising your manuscript, if you decide to resubmit it to eNeuro or to another journal.

-----------

REVIEWER #1

ADVANCES THE FIELD

The article builds a bridge between the literature focusing on the role of oscillations in neural information processing and literature focused on neural mechanisms of object perception, which is dominated by fMRI studies (and invasive recording studies in monkeys).

COMMENTS

This study performs a set of novel analyses on an existing dataset published by Cichy et al., (2014), consisting of fMRI and MEG measurements made while healthy participants viewed a set of object images. In that previous paper, fMRI responses in two visual brain regions (V1 and IT) were linked to responses measured with MEG through representational similarity analysis, whereby the MEG RDMs were created by performing decoding analysis on the raw (or lightly preprocessed) MEG timeseries. It was found that MEG activity early after stimulus onset corresponded to V1 while later MEG activity corresponded to IT, although there were also ‘sustained’ correlations with V1. In the current manuscript, the RDMs are derived from that same MEG data but RDMs were created after separating the data into distinct oscillatory components (time-frequency decomposition) to investigate if specific ‘parts’ of time-frequency space are primarily linked to fMRI responses in either V1 or IT. These MEG TF RDMs are also compared to different layers of set of deep neural networks. From this analysis, ‘a complex picture’ emerges, in which early/low-level TF components of processing are not necessarily all tied to V1 and TF late/high-level components are not all tied to high-level processing in IT. This speaks against a model of object recognition which consists of a single, one-directional pass through a feedforward brain network.

The authors motivate the study by suggesting that there is gap in the literature between research focusing on oscillatory brain signals and research on object representations in different visual brain regions. I quite agree with their assessment and therefore I think the study has potential to provide a nice contribution to the existing literature. However, the presented set of analyses are quite complex and removed from the data: there are many transformations between the recorded data and the final results, and it’s not clear whether the authors went into this having clear theoretical predictions about which oscillatory components should reflect specific aspects of object perception (or not) or how one should interpret, say, a correlation with stimulus dissimilarity reflected in a ‘phase’ versus an ‘power’ component. All in all, the final conclusion (‘it’s complex’) seems a bit weak/disappointing after taking the readers through such an elaborate analysis. I think it is critical that the authors provide more explicit theoretical predictions and interpretations of how their oscillatory measures relate to the neural processing that takes place in the underlying circuits and how this gives rise to object recognition. In addition, some aspects of the analysis need to be explained a bit better. I elaborate on these points in more detail below.

Major comments:

* What does this paper add beyond what has been shown in the Cichy et al. papers on the same data (both the MEG/fMRI fusion paper and the DNN comparison)? They also showed early correlations with V1 and late correlations with IT but also sustained correlations with V1. What insight does the oscillatory analysis ‘add’ to the functioning of the visual system other than ‘it’s complicated’, or ‘there’s probably feedback’? The paper would benefit from more explicit theory about how oscillations contribute encoding of visual stimuli. For example, in the Discussion section, the sentence describing ‘the picture that emerges’ (page 10) is just a very broad statement that there a lot of different things going on. How do the authors think that phase and power signals can ‘carry representations’? How do the current results illuminate that question?

* The spaces we are looking at in this paper are quite removed from the data: there are many transformations and it’s easy to lose track of that is being measured and how to interpret that measure. If I understood correctly, it essentially boils down to ‘a correlation of a cluster centroid RDM based on RDMs on a TF representation of MEG signals’. While I understood the logic behind most of these transformations (except perhaps the clustering analysis; see next comment), I would also like authors to establish the link with the underlying neural signals more throughout: what does ‘sustained phase’ mean in the context of RSA? For example, is the hypothesis that neurons within neural circuit in V1 or IT become more aligned when a stimulus comes on, and then they maintain this more for some stimuli than for others? Conversely, I think that an RDM based on power differences shows that the oscillation has more amplitude for some images than for others: but why would that be the case? What does it ‘mean’ if the power or phase across images is more similar somewhere in TF space? In other words, the results need to be coupled back to the original data and neural processes more, and better guidance on to interpret results based on RSA on these TF transformed data is needed.

* I had some trouble understanding the clustering analysis. I understand this analysis was done to reduce the dimensionality of the TF/RDM space, but I didn’t quite follow why it then again yields in a separate power/phase TF map (Figure 5/6), given that it is conducted in the combined power/phase space? Also why is the ‘distance to the centroid’ the measure of interest in these maps; is that the centroid of each cluster itself, or of all the clusters combined (measuring some kind of distinctiveness of the cluster?). Even though the analysis is spelled out in terms of the number of data points that go into it and number of clusters that were picked, I think I’m just missing a key point of the logic, and I think it would be helpful if the authors could provide a figure analogous to for example Figure 1 (and also Figure 2, which is also a Methods figure) explaining the analysis step by step, in addition perhaps to providing some (pseudo)code.

* I liked how the authors attempted to clarify often conflated terms in the literature such as early/late and low-/high-level. I had trouble however understanding what they meant with the third distinction, ‘basic’ and ‘refined’, which is a distinctive qualifier they assigned to different DNN layers (lower layers vs higher layers). I don’t really understand the basis for that distinction. I understand why you would want to not automatically assume that low vs. high layers in DNNs correspond to low vs. high regions in the brain, but the word ‘refined’ just seems very vague. What does ‘refined’ mean? Is it more semantic, more object-like, more label-like (as in the DNN output label), less pixel-like? Why is it the case that ‘Performing RSA between MEG oscillatory RDMs and DNN layer RDMs should reveal which features of the MEG oscillatory representations correspond to basic vs. refined object representations’ (page 10)? Why should we assume that the MEG representation becomes more refined over time?

* DNNs perform many repeated convolutions using increasingly larger pooling regions. The MEG signals are derived from mass activity of large populations of neurons that presumably also combine information across the visual field. Could this inherent ‘smoothing’ of the visual representation through convolution in higher layers somehow bias the RSA correlations? In the sense that you are always more likely to find correlations with higher layers for these types of data? This is perhaps somewhat evidenced by the data presented in Figure 6, for the clusters reflecting pre-stimulus activity: according to the authors, these data ‘cannot possibly encode the identity of a stimulus that has not been presented yet’ (Page 10). Yet most of the correlations are with mid- to high-level DNN layers. How should we interpret these correlations then?

* The choise to include results for these pre-stimulus clusters in Figure 6 was also a bit puzzling to me, especially if the authors indeed decided to ‘not investigate further’ the prestimulus activity related clusters (as claimed earlier on in the Results). If these signals are not informative about object processing, then why should we look at their correlations with DNNs and fMRI RDMs - are we just looking at noise here? Are these correlations less significant than for the post-stimulus representations, and if not, how do we then know that we can trust the correlations for the data presented in Figure 5?

* I wondered how to square the lack of correlation of the MEG RDMs with low-level DNN layers with other MEG RSA studies that have shown strong correlations with low-level DNN layers, looking at the RDMs based on decoding of the raw signal (e.g. Bankson et al., 2018, Figure 5/6). Rather than seeking an explanation in terms of the neural process itself (as discussed on Page 12), could this be due to an analysis choice, e.g. the normalization across layers, or the TF decompositions?

Minor comments:

Page 1 (bottom): “the different levels of object representation in the brain”: what are those levels?

Page 4 (methods): It might be helpful to refer to ‘condition’ as ‘stimulus condition’ (to distinguish from some kind of task manipulation or measurement condition).

Page 4 (methods): ‘This procedure resulted in four RSA maps’ It might be helpful to explicitly say this is 4 maps per subject (since the fMRI RDMs are averaged across subjects before comparison).

Page 5 (methods): ‘The chosen clusters could be visualized...’ This is the part of the cluster analysis I don’t quite understand, please elaborate more on this part.

Page 5 (methods): Eq 1: I assume that R stands for correlation and I for something like ‘information’ or ‘index’ but all terms of an equation should always be explicitly explained.

Page 5 (methods): ‘... and the number of iterations was counted’, I think you mean proportion or percentage?

Figure 2: the tSNE embedding method is not explained in the methods.

Figure 2: it would also be nice to show the distribution of images after recentering (so readers can determine if it lead to a distortion of the space).

Page 7 (results): ‘The advantage of measuring partial correlation...’ I get this point, but it could still be good to report full correlations along with the partial correlations, so readers can get a sense of the magnitude of the correlations and also to compare with the prior literature where V1 or IT ROIs were matched individually.

Figure 3: the lack of a significant correlation of gamma power with IT is striking to me. This seems to align with recent work by Hermes et al., (2015) who found that gamma oscillations appear to be particularly elicited by stimuli with a specific structure (like gratings), not most other stimuli (like the objects used here).

Figure 3: I’m wondering how to interpret the clear broadband effect in the Phase-V1 correlation map (panel c). Does this reflect an alignment of the oscillations across frequencies due to stimulus onset? What if you would subtract the ERP from the data before doing the TF decomposition; would this remove this effect?

Page 7 (results): In the paragraph about latency differences, make sure the wording always consistently refer to the results as ‘correlations’; these data can’t actually speak to an ‘onset response in’ V1, or effects that are not observed ‘in IT’; it’s all based on correlations between a non-temporally varying fMRI response and MEG.

Page 7 (results, final sentence on page): I had trouble understanding what ‘a sustained oscillatory phase resembling IT representations’ means (see also main comments).

Page 8: ‘To evaluate the relative importance...’ this sentence seems agrammatical.

Figure 5: In the text it is claimed that cluster 1 is more V1-like than cluster 3, but the two insets showing the relative V1/IT correlation for panels a and c look identical to me.

Page 10: ‘Despite slight differences between the four DNNs...’ in many of the insets in Figure 5 those differences between DNNs actually look quite substantial to me. Perhaps more data could be shown (e.g. the each DNNs correlations pre-normalization) to make this claim more convincing.

Page 10: ‘Based on the logic above, this cluster is likely to reflect feedback signals...’ I kind of understand the logic but I wasn’t totally convinced. For example, why would this feedback signal be linked to sustained beta-gamma power rather than another frequency? Why is it only reflected in the power and not the phase? Again, it would help if there was more theoretical framing around the role of oscillations in e.g. feedback.

Page 11 (discussion): ‘Importantly, the representational dynamics of brain oscillations can be very differently expressed...’ It could be helpful to include a (cartoon) figure depicting why you would observe such differences and what they mean: some sort of figure depicting the different stages of object processing and how they are supported by the different oscillatory components, i.e. a visual representation of the main conclusion.

General comment: As a reviewer it would be helpful to have line numbers and to have figures be positioned more closely to the text that describes them.

-----------

REVIEWER #2

ADVANCES THE FIELD

The results strengthen the understanding of the visual content of different MEG spectral power and phase signals, in relation to V1 and IT fMRI activity and to feedforward convolutional neural networks. The most striking finding was the discovery that sustained beta-gamma oscillatory power in V1 (‘cluster 3’) was correlated with “high level", not “low level", representations as gauged by deep neural networks. Many previous reports have indicated that gamma power in early visual areas, particularly in superficial layers, is consistent with complex representations and attention, but this manuscript addresses this topic in more detail by separating power and phase and by testing 4 different DNN models. Its use of MEG may have also improved sensitivity to weak signals compared to previous studies based on other methods.

COMMENTS

The results strengthen the understanding of the visual content of different MEG spectral power and phase signals, in relation to V1 and IT fMRI activity and to feedforward convolutional neural networks. The most striking finding was the discovery that sustained beta-gamma oscillatory power in V1 (‘cluster 3’) was correlated with ‘high level", not “low level", representations as gauged by deep neural networks. Many previous reports have indicated that gamma power in early visual areas, particularly in superficial layers, is consistent with complex representations and attention. A recent report using intracranial depth recordings has linked gamma power in lower visual areas to object content in the lower DNN layers, gamma power in higher visual areas to higher DNN layers, and the representational geometry of neural activity in the theta range (5-8 Hz) to higher DNN layers (Kuzovkin et al 2018 Communications Biology). The discrepancy between this manuscript’s results and those of Kuzovkin et al is interesting and could be discussed. Perhaps they are due to the differences in approach, e.g. this manuscript’s use of representational dissimilarity matrices and k-means clustering, which may be able to extract weaker signals from more evenly and broadly sampled locations across the brain, compared to Kuzovkin et al’s fewer number of intracranial electrodes per patient. This manuscript’s testing of 4 different types of DNNs may have also improved the generalizability of the comparison to DNNs.

This manuscript also advanced the science by examining the object content based on phase information, revealing an interesting association between broadband phase activity with the stimulus turning off (cluster 4) and with lower DNN layers, and stimulus turning on (cluster 1) with mid-level DNN layers. This appears to be consistent with ON/OFF responses in V1 (Jansen et al... J-M Alonso 2019 Cereb Ctx).

Overall I think the quality of the advances is sufficient, though I would recommend a few changes:

1) It would be helpful to show examples of the stimuli shown.

2) The discrepancy between the 500 ImageNet images and 92-image stimulus set wasn’t clear, i.e. why would the 92-image set be localized to one corner of the t-SNE space. The authors hint that this may be due to a difference in image backgrounds. This claim could be strengthened by showing insets of stimuli from the larger set whose t-SNE positions are interspersed among the t-SNE positions of the 92-image stimulus set, as well as some that are more distant. This could also address #1.

Minor:

3) The authors alternately refer to RSA and to RDM. It might be clearer to refer to RDM more consistently in the text.

4) Results from GoogleNet and AlexNet appeared to be more extreme than those of the other DNNs. It might be worthwhile to speculate briefly in the Discussion what this might be so. The ‘cornerness’ of the 92-image stimulus set was shown for VGG layer 6 and layer 12. Was this true as well for the other layers and other DNNs? Perhaps, instead of showing both layer 6 and layer 12, it would be more helpful to use half of that space to plot ‘cornerness’ vs layer for the different DNNs. This might also help explain some of the variability across DNNs.

5) Although the generalizability of these results appears to be adequately addressed, there are potential issues that cannot be easily addressed with this paradigm. For example, microsaccades may have contributed to some of the MEG variability across objects, and the results with these particular spectral frequencies may not generalize to other tasks that are not passive viewing of sequentially presented images.
